# Face memory and facial expression recognition are both affected by wearing disposable surgical face masks

**DOI:** 10.1007/s10339-022-01112-2

**Published:** 2022-10-15

**Authors:** M. Ventura, A. Palmisano, F. Innamorato, G. Tedesco, V. Manippa, A. O. Caffò, Davide Rivolta

**Affiliations:** Department of Education, Psychology and Communication, University of Bari Aldo Moro, Bari, Italy

**Keywords:** Face memory, Face perception, Emotion recognition, Face masks, Disposable surgical face masks (DSFMs)

## Abstract

Faces are fundamental stimuli for social interactions since they provide significant information about people’s identity and emotional states. With the outburst of the COVID-19 pandemic, global use of preventive measures, such as disposable surgical face masks (DSFMs), has been imposed. The massive use of DSFMs covering a large part of the face could interfere with identity and emotion recognition. Thus, the main aim of the current study was (i) to assess how DSFMs affect identity recognition (Experiment 1), (ii) how DSFMs affect emotion recognition (Experiment 2), and (iii) whether individual empathy levels correlate with emotion recognition with DSFMs. The potential relation between identity and emotion recognition with and without DSFMs was also investigated. Two tasks were administered to 101 healthy participants: (i) the *Old-new face memory task* aimed to assess whether the learning context (i.e., DSFMs on/off) affects recognition performance, whereas (ii) the *Facial affect task* explored DSFMs’ effect on emotion recognition. Results from the former showed that the stimuli’s features in the learning stage affect recognition performances; that is, faces wearing DSFMs were better recognized if wearing DSFMs at first exposure and vice versa. Results from the *Facial affect task* showed that DSFMs lead to reduced disgust, happiness, and sadness recognition. No significant correlation emerged between identity and emotion recognition. The Interpersonal Reactivity Index (IRI) was administered to assess affective and cognitive empathy; however, IRI scores did not correlate with either face memory recognition or facial affect recognition. Overall, our results demonstrate (a) a “context effect” for face memory with and without DSFMs; (b) a disruptive effect of DSFMs depending on the expressed emotion; and (c) no correlation between empathy and emotion recognition with DSFMs.

**Supplementary Information:**

The online version contains supplementary material available at 10.1007/s10339-022-01112-2.

## Introduction

Faces represent the stimuli we rely on the most for social interactions, since they convey information about other people’s identity, emotion, attractiveness, age, and gender. Much research has demonstrated that typical face perception occurs via face-specific visual mechanisms defined as “holistic,” which refers to the recognition of faces as a whole, rather than a sum of individual face parts (McKone et al. 2009; Bossi et al. 2020; Bonemei et al. 2018; Negrini et al. 2017). It follows that impaired holistic processing characterizes atypical face perception in clinical conditions (e.g., Prosopagnosia) (Rivolta et al. 2014; Palermo et al. 2011; Monti et al. 2019), and autism spectrum disorder (ASD) (Webb et al. 2017).

Previous research has also shown that holistic processing can be disrupted by various forms of concealments, resulting in altered face memory performances. For instance, Patterson and Baddeley (1977) investigated whether face memory was affected by major changes in face appearance (wigs, glasses, fake mustaches, and beards) in an Old-new discrimination task; their results showed that disguise manipulations reduce recognition accuracy almost to chance level. Studies using face-matching tasks demonstrated that face perception is reduced when faces are occluded with, for instance, sunglasses (Graham and Richie 2019; Kramer and Richie 2016), ski masks (Manley et al. 2019), or masks made from nylon stockings (Mansour et al. 2020). This has strong implications in security and forensic contexts based on face recognition as a method for offenders’ identification, since face coverings negatively impact eyewitness identifications accuracy in line-ups (Shapiro and Pendrod 1986). Importantly, the identification accuracy improves when the line-up matches facial features at encoding (e.g., masked line-up if perpetrator was masked) compared to unmatched encoding and retrieval (Davies and Flin 1984; Manley et al. 2019).

The research reviewed above is of great relevance for the current COVID-19 pandemic, which imposed the global adoption of preventive measures, such as the use of disposable surgical face masks (DSFMs) in public places. As such, DSFMs have the potential to disrupt people’s face recognition skills and negatively impact social interactions (Ferrari et al. 2021). In line with this hypothesis, Carragher et al. (2020) showed a detrimental effect of DSFMs in judging whether two simultaneously presented faces (i.e., Glasgow face-matching task—GFMT; Burton et al. 2010) depicted the same person or two different (familiar/unfamiliar) people. Freud et al. (2020, 2021) found that DSFMs lead to a strong decrease in performances at the Cambridge face memory test (CFMT) (Duchaine et al. 2006) assessing memory for unfamiliar faces; moreover, this reduction was accompanied by a smaller “Inversion effect” (Farah et al. 1995), suggesting that DSFMs may disrupt holistic mechanisms. Furthermore, Noyes et al. (2021) who tested DSFMs and sunglasses’ effects on familiar/unfamiliar identity recognition on a face-matching task concluded that face occlusion leads to reduced accuracy in face recognition, albeit with sunglasses showing a smaller impact as compared to DSFM. An aspect that has never been explored, and that has potential theoretical and practical implications, is whether a “learning context effect” occurs: are we better at recognizing faces with DSFMs if we learn them with DSFMs? Do DSFMs always cause a detrimental effect on face recognition? Furthermore, the role of DSFMs in emotion perception still remains controversial.

Much evidence from human neuroimaging (Haxby et al. 2000), patient populations (Duchaine et al. 2003; Duchaine et Garrido 2006; Duchaine et al. 2003), and cognitive psychology (Bruce and Young 1986) demonstrates that facial identity and facial expressions of emotions rely on separate anatomical routes and cognitive mechanisms. This makes it interesting to investigate whether DSFMs negatively affect face identity and/or facial expression recognition. Theories of emotion perception suggest that specific facial *features*, such as eyes and mouth, are critical to distinguish each expression from all others (Calvo and Nummenmaa, 2008; Wegrzyn et al. 2017). However, given that emotion recognition is affected by visual manipulations, such as the Composite-face effect (Calder et al. 2000) and the face inversion effect (Derntl et al. 2009), *holistic* mechanisms also play a critical role in facial expression recognition (Derntl et al. 2009a, b; Calder et al. 2000; Tanaka et al. 2012; Palermo et al. 2011). By hiding the bottom-half of face stimuli, thus, DSFMs can be useful to explore perceptual mechanisms implied in facial expression recognition.

Previous evidence demonstrated that occluding the face, even partially, makes emotion recognition harder, as in the case of the Islamic headdresses, especially burqa (Kret and de Gelder 2012), facial eyes occlusion with sunglasses or virtual reality glasses, and facial mouth occlusion with DSFMs or scarves (Kotsia et al. 2008). Several recent studies explored the role of DSFMs in human emotion perception, and results are controversial. Marini et al. (2021) reported decreased emotion recognition rates for happiness, fear, and sadness with standard DSFMs as compared to transparent masks (which leaves the mouth visible) and no-DSFMs conditions; Grundmann et al. (2021) found an overall decrease in recognition accuracy for the basic emotions when face stimuli were presented with DSFMs, while Calbi et al. (2021) demonstrated that DSFMs and face scarves have no detrimental effect on anger and happiness recognition. Gori et al. (2021) examined the impact of DSFM on different age group (toddlers, children, and adults) for fear, happiness, sadness, and anger, reporting a general performance decline in all age cohorts, but highlighting that toddlers’ performance is more affected by DSFMs than children’s and adults’. On a different note, Grenville and Dwyer (2022) showed that DSFM effect on emotion recognition varied across emotions, with some of them having a clear advantage without DSFM and others (i.e., anger and fear) paradoxically being better detected with DSFM. The inconsistencies observed in the outlined literature require further investigation, not solely for the practical relevance of the issue, but also from a theoretical perspective.

A significant amount of research has also investigated whether being able to accurately recognize emotions is mediated by “being empathic” (Davis 1994). Several studies examined the relationship between empathy and facial expression recognition ability, highlighting positive relationships between self-reported emotional empathy and facial expression recognition (Besel and Yuille 2010). It might be argued that individual differences in empathy may mediate the recognition of others’ emotions, even when they wear DSFMs. The “empathy” construct is made of two components: the affective response to another person and the cognitive capacity to intentionally take others’ perspectives (Decety and Jackson, 2006). Specifically, Davis (1983, 1994) identified 4 constitutive abilities of cognitive and affective empathy: perspective taking (the ability to adopt another person's point of view), fantasy (the tendency to imagine oneself in fictitious situations), empathic concern (the ability to experience feelings of warmth and concern for others), and personal distress (the tendency to experience feelings of anxiety and discomfort as a result of another’s negative experience). It is possible that high empathy scores could help people to properly recognize emotions even when face parts are hidden.

Based on potential dissociations between identity and emotion perception, in the current study we aimed to (i) explore potentially atypical face memory in relation to the use of DSFMs (at learning and test) and (ii) explore the characteristics of facial expression recognition difficulties when faces wear DSFMs. The potential correlation between individual empathy levels and emotion recognition when faces are occluded by DSFMs was also investigated. Two experiments were run to investigate the effects of DSFMs on visual cognition; Experiment 1 tested face memory with and without DSFMs with an *Old-new face memory task*, whereas Experiment 2 assessed facial expression recognition with and without DSFMs via the *Facial affect task*. We hypothesized that DSFMs negatively impact face, as well as emotions recognition accuracy, and that higher dispositional empathy levels, as measured via the Interpersonal Reactivity Index (IRI) (Davis, 1980), correlate with emotion recognition when faces are partially covered by DSFMs.

## Methods

### Participants

A total of 101 participants (66 F; age range: 18–49; mean age: 23.7) were recruited via *snowball sampling* during the period of April–May 2021. To check for the appropriateness of the sample size, G*Power analysis for repeated measures ANOVA was conducted and reported a total sample size of 20 and 35 participants for Experiments 1 and 2, respectively.^1^ However, to more confidently have good experimental power, and in line with other recent research on the topic (~ 100 in Calbi and colleagues 2021), our sample size was bigger than the sample indicated by G*power.

To test emotion and identity recognition ability, two independent tasks were programmed with the PsyToolkit platform (Stoet 2010) and administered in a counterbalanced order (see below for tasks description). All participants provided informed consent before completing the experiments. For the assessment of dispositional empathy, participants completed the IRI (Davis 1980). Training sessions were also administered to assure familiarization with the tasks. Due to the pandemic-related restrictions, both experiments were run remotely (i.e., via a one-time accessible link). To check on the proper execution and fulfillment of the tasks, participants were asked to share their computer screen with the researchers, except while completing the IRI to avoid potential social desirability bias.

### Experiment 1—old-new face memory task

#### Materials and procedure

**Fig. 1:**
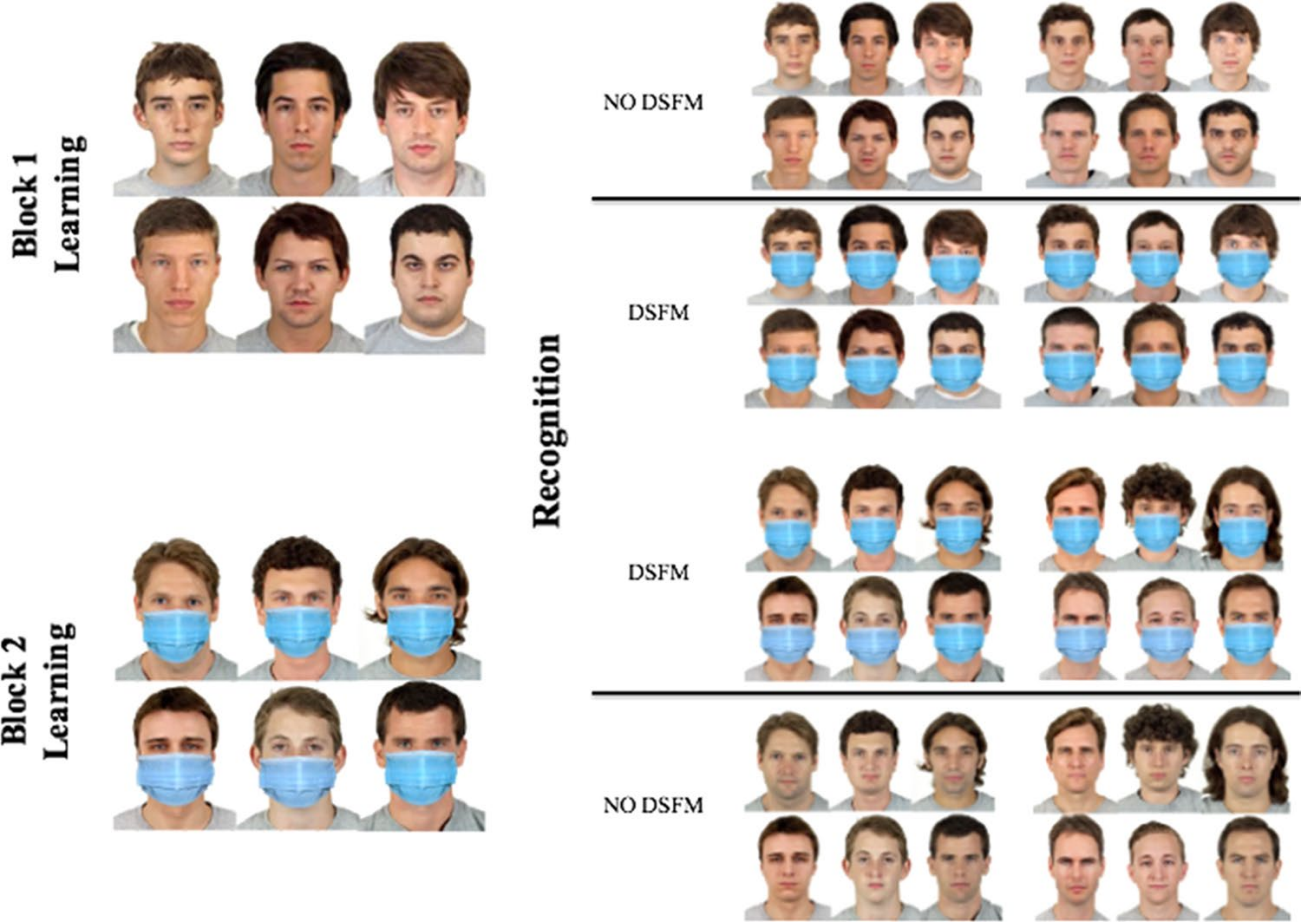
*Old-new face memory task.* Stimuli from Experiment 1. Block 1 consisted of learning phase without DSFMs and test with and without DSFMs. Block 2 consisted of a learning phase with DSFMs and a test with and without DSFMs. Participants had to pick out which faces they saw during the learning phase

To assess face recognition and learning with and without DSFMs, an *Old-new face memory task* was administered. The task consisted of 2 blocks, each composed of a learning and an actual testing phase. In the learning phase of Block 1, 6 face stimuli without DSFMs were simultaneously presented, and participants were asked to memorize them in 30 s. Afterward, in the testing phase, participants had to recognize the 6 previously shown identities among 6 distractors by answering, for each face, if it was one of the previously learned or not. Each stimulus in the testing phase was displayed twice, both with and without DSFMs, for a total of 24 trials. Block 2 had the same structure as Block 1. However, new identities were used (both in the learning and test phase) and faces in the learning phase were presented with DSFMs (see Fig. 1). The stimuli were selected from the “Chicago Face Database 2015” (Ma et al. 2015) and presented in colors, in the foreground, and with a white background. Features from the original pictures such as hair and ears were not altered to ensure the ecological validity of the experiment. Moreover, all the selected face stimuli were from Caucasian male adults, to avoid ethnic, age, and gender biases (Wang et al. 2014; Wang 2013; Hall and Matsumoto 2004). DSFMs were digitally added to the stimuli using the “MaskOn your profile for Covid-19 Safety” (Kapwing 2021) since artificially imposed mask does not have a misleading effect compared to natural posed ones (Grenville and Dwyer 2022) (Fig. 1). Before starting the task, participants completed a training session (with face stimuli not included in the actual experiment) to familiarize themselves with the procedure.

#### Data analysis

Data from the *old-new face memory task* were analyzed according to the principles of Signal Detection Theory (Green and Swets 1966). Albeit accuracy is a good indicator of the overall performance, *d’* (i.e., a measure of sensitivity) is a better index of recognition discriminability since it takes into account false alarms. Specifically, *d*’ was calculated by subtracting the z scores for false alarm (FA) responses from z scores for correct responses (hits − H) [d’ = z(H) − z(FA)] (Stainslaw and Todorow 1999), where increasing values of *d’* refer to a greater sensitivity to a given signal. Response bias was calculated as the β value. This is obtained with the following equation: ln(ß) = *cd’* = − 1/2 [z(H)^2^ − z(FA)^2^]. An observer who is maximizing H while minimizing FA will have a ß that is equal to 1.00 (i.e., no bias). A value of ß below 1.00 represents a liberal tendency, i.e., to report most of the times that the target is present, while a high value of ß 1 (i.e., above 1.0) represents a conservative tendency, i.e., to report most of the times that the target is absent (Gardner et al. 1984). These procedures were implemented for both tasks.

A repeated measures ANOVA was calculated on *d*’ and β scores with two factors: “learning” (without DSFMs Vs. with DSFMs) and “test” (face recognition without DSFMs Vs. face recognition with DSFMs). Post hoc analyses were Bonferroni corrected. *d*’ and β calculations were carried out using R’s psycho package (vo. 6.1.; Makwoski 2018), while ANOVA and post hoc analyses were carried out using SPSS (Version 26.0).

#### Results

**Fig. 2:**
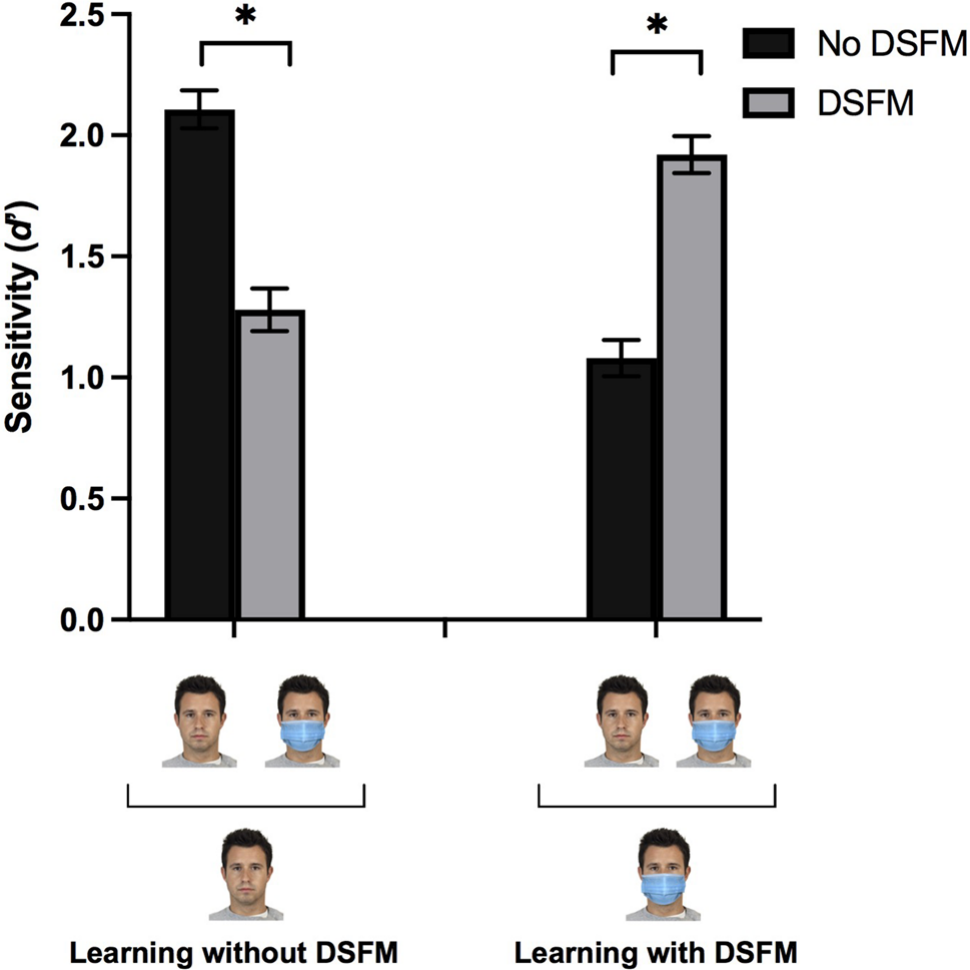
Mean sensitivity (d') for No DSFMs (black) vs. DSFMs (gray) condition for Block 1 and Block 2

The repeated measures ANOVA showed a main effect of “learning” [F(1,100) = 6.68, *p* = 0.011, *η*^2^_*p*_ = 0.063], where learning without DSFMs (M = 1.7, SEM = 0.07) led to a better performance than learning with DSFMs (M = 1.5, SEM = 0.06). There was no main effect of “test” [F(1,100) = 0.11, *p* = 0.916, *η*^2^_p_ = 0.000]. There was a statistically significant interaction between learning and test [F(1,100) = 216.32, *p* < 0.001, *η*^2^_p_ = 0.684]. Post hoc comparisons (Fig. 2) indicated that recognition without DSFMs (M = 2.1, SEM = 0.08) was higher than recognition with DSFMs (M = 1.28, SEM = 0.08) when the study faces were presented *without* DSFMs (*p* < 0.001, *η*^2^_*p*_ = 0.512) (Block 1). However, recognition without DSFMs (M = 1.08, SEM = 0.07) was lower than recognition with DSFMs (M = 1.9, SEM = 0.07) when the study faces were presented *with* DSFMs (*p* < 0.001, *η*^2^_*p*_ = 0.517) (Block 2).

As for the repeated measures ANOVA on ß, the main effect of test was statistically significant [F(1,100) = 3.97, *p* = 0.049, *η*^2^_*p*_ = 0.038], while the main effect of learning was not [F(1,100) = 0.393, *p* = 0.532, *η*^2^_*p*_ = 0.004). The interaction between learning and test was statistically significant [F(1,100) = 25,89, *p* < 0.001, *η*^2^_*p*_ = 0.206], with post hoc comparisons showing that ß of trials without DSFMs (M = 1.13, SEM = 0.04) was higher than that with DSFMs (M = 1.01, SEM = 0.03) when the study faces were presented *without* DSFMs (*p* = 0.026, *η*^2^_*p*_ = 0.048). When study faces were presented *with* DSFMs, ß of trials without DSFMs (M = 0.97, SEM = 0.03) was lower than that with DSFMs (M = 1.22, SEM = 0.04) (*p* < 0.001, *η*^2^_*p*_ = 213).

Results, thus, demonstrated that it is easier for participants to recognize faces without DSFMs only if the same faces are learnt without DSFMs; by contrast, they find it easier to recognize faces with DSFMs if they were first exposed to them with DSFMs. Moreover, results from ß showed that the response bias of with/without DSFMs trials reflects the learning modality (i.e., higher ß with DSFMs when learning was with DSFMs, and vice versa). Thus, there might be a “context effect” between learning and test, which implies that DSFMs are not always disruptive.

### Experiment 2—facial affect recognition

The second experiment aimed to investigate participants’ ability to recognize emotions with and without DSFMs. Specifically, the experiment aimed at (i) verifying whether DSFMs negatively impact emotions recognition and (ii) examining a possible correlation between emotion recognition (with and without DSFMs) and empathic abilities.

#### Materials and procedure

The *Facial affect task* consisted of 60 stimuli of Caucasian faces, selected from the Karolinska Directed Emotional Faces (KDEF) database (Lundqvist et al. 1998). The “MaskOn your profile for Covid-19 Safety” software (Kapwing 2021) was adopted to digitally add DSFMs to half of the selected faces. The stimuli included 6 different male identities for each basic emotion (disgust, fear, happiness, sadness, anger); each face was consequently presented 5 times, both with and without DSFMs, for a total of 60 trials. Surprise was excluded because of its hedonically neutral connotation (Reisenzein et al. 2019).

**Fig. 3:**
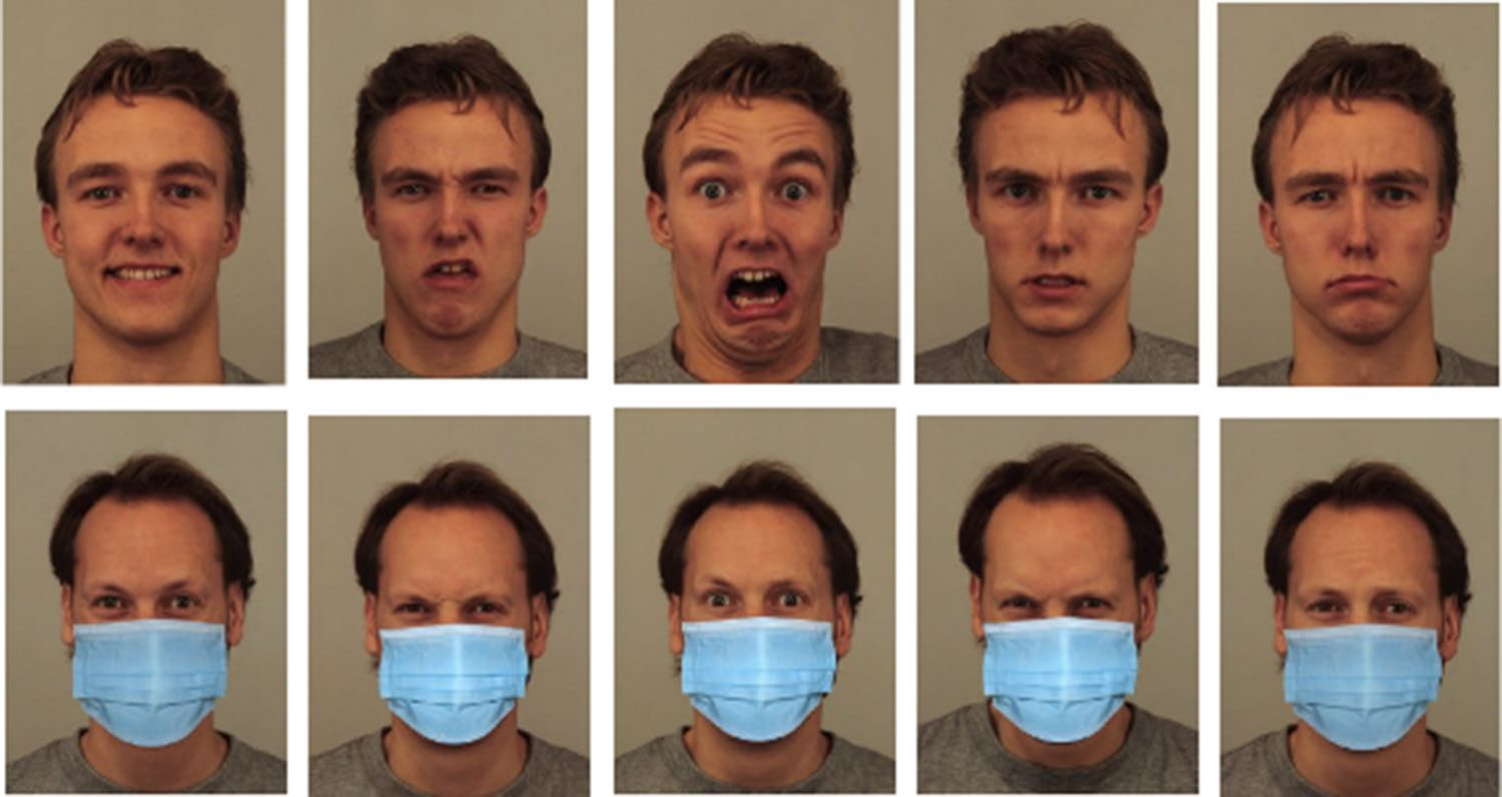
*Facial affect task.* Sample stimuli from Experiment 2. Each basic emotion (happiness, disgust, fear, anger, and sadness) was presented with and without DSFMs

Stimuli were displayed for 3.5 s, and then, participants had 10 s to label the emotion by choosing within a list of 5 options (anger, disgust, fear, sadness, and happiness) (Fig. 3). The time limit set for both presentation and recognition is aimed at simulating everyday life situations in which emotional expressions are perceived in a few seconds (Tracy and Matsumoto 2008) (i.e., typically between 0.5 to 4 s) (Ekman 1993). After completing the Emotion recognition task, participants were asked to fill the Italian version of Davis's IRI (Albiero et al. 2006), a widely used measure of cognitive and affective empathy including four different domains (Perspective Taking, Fantasy, Empathic Concern, and Personal Distress) (Davis 1980).

#### Data analysis

A repeated-measures ANOVA was performed on *d’* scores, including two factors: “emotion” (disgust, fear, happiness, sadness, and anger) and “condition” (without DSFMs vs. with DSFMs). However, for *d’* calculation, we considered each emotion separately, for both conditions (without and with DSFMs). For each participant, a DSFMs Performance Index was calculated for each emotion by subtracting the *d’* scores of the emotion recognition with DSFMs from those without DSFMs, to obtain a metric of costs and advantages of DSFMs. The potential relationship between emotion recognition and empathy was assessed by running *Pearson’s correlations* between DSFMs.

#### Results

**Fig. 4:**
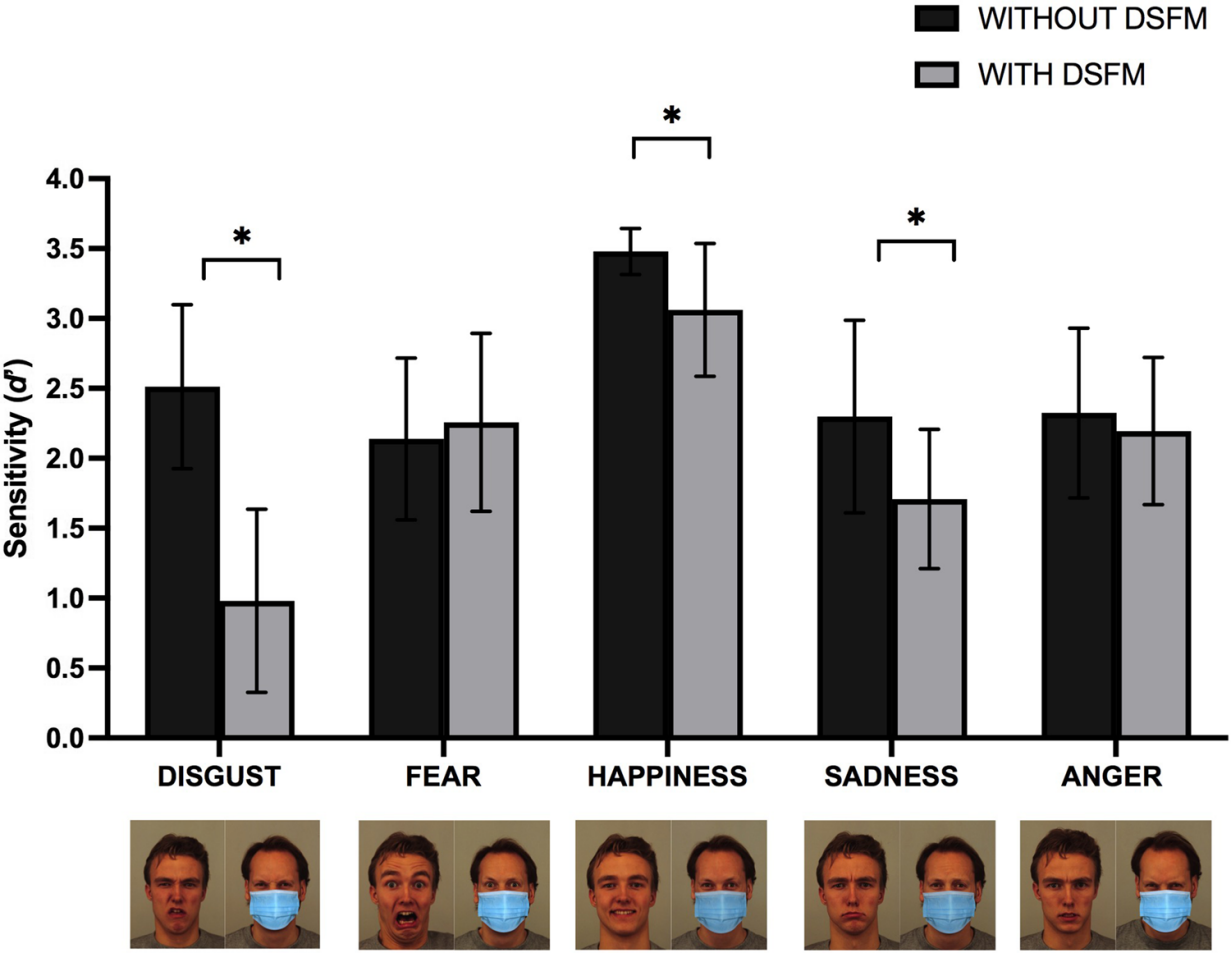
Mean sensitivity (d') or no DSFMs (black) vs. DSFMs (gray) condition for each basic emotion

Repeated measures ANOVA showed a main effect of DSFMs, with facial expressions without DSFMs (M = 2.55, SEM = 0.03) being better recognized than with DSFMs (M = 2.04, SEM = 0.03) [F(1,100) = 146.73, *p* < 0.001, *η*^2^_*p*_ = 0.594]. A main effect of “emotion” [F(4,400) = 274, *p* < 0.001, *η*^2^_*p*_ = 0.930] showed that disgust recognition (M = 1.74, SEM = 0.04) is lower than fear (M = 2.2 SEM = 0.04), happiness (M = 3.27, SEM = 0.026), sadness (M = 2, SEM = 0.04), and anger (M = 2.25, SEM = 0.04); fear is less accurately recognized than happiness and anger, but it is better recognized than sadness; happiness recognition is higher than sadness and anger and, finally, sadness recognition is lower than anger. There was also a statistically significant interaction between emotion and DSFMs [F(4,400) = 100.6, *p* < 0.001, *η*^2^_*p*_ = 0.795]. Post hoc comparisons indicate that disgust recognition was higher without DSFMs (M = 2.5, SEM = 0.05) than with DSFMs (M = 0.98 SEM = 0.065) (*p* < 0.001, *η*^2^_*p*_ = 0.779); similarly, happiness recognition was higher without DSFMs (M = 3.5; SEM = 0.01) than with DSFMs (M = 3; SEM = 0.04) (*p* < 0.001, *η*^2^_*p*_ = 0.437), and sadness recognition was higher without DSFMs (M = 2.3; SEM = 0.06) than with DSFMs (M = 1.7, SEM = 0.05), (*p* < 0.001, *η*^2^_*p*_ = 0.393); finally, no significant differences were found for fear recognition without (M = 2.13, SEM = 0.05) and with DSFMs (M = 2.25 SEM = 0.06) (*p* = 0.114, *η*^2^_*p*_ = 0.025), and anger recognition without DSFMs (M = 2.32, SEM = 0.06) and with DSFMs (M = 2.19, SEM = 0.052), (*p* = *0.0*73, *η*^2^_*p*_ = 0.032) (Fig. 4).

The repeated measures ANOVA on ß showed a significant main effect of emotion [F(4,400) = 5.63, *p* > 0.001, *η*^2^_*p*_ = 0.179], main effect of condition [F(1,100) = 89.69, *p* < 0.001, *η*^2^_*p*_ = 0.473], and a significant interaction between emotion and condition [F(4,400) = 47.89, *p* < 0.001, *η*^2^_*p*_ = 0.639]. Post hoc contrasts on the interaction between emotion and condition interaction showed that ß was significantly different between disgust without DSFMs (M = 1.49, SEM = 0.104) and disgust with (M = 3.04, SEM = 0.216) (*p* < 0.001, *η*^2^_*p*_ = 0.302), sadness without (M = 3.92, SEM = 0.231) and with DSFMs (M = 2.45, SEM = 0.115) (*p* < 0.001, *η*^2^_*p*_ = 0.269), and anger without DSFMs (M = 4.45, SEM = 0.25) and with DSFMs (M = 1.24, SEM = 0.09) (*p* < 0.001, *η*^2^_*p*_ = 0.602). These results show a higher response bias without DSFMs for sadness and anger, while the opposite occurs for disgust, and no significant bias between conditions emerges for happiness and fear.

**Fig. 5:**
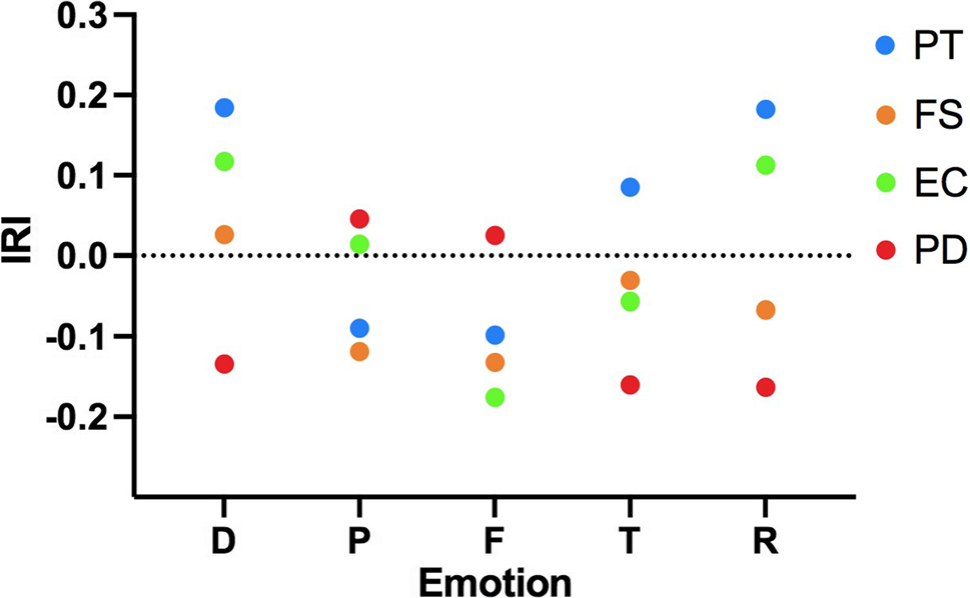
Scatterplot for participants’ DSFMs Performance Index and IRI correlations

Participants’ DSFMs Performance Indexes (see Paragraph 2.3.2) were correlated with the IRI scores. The means and standard deviations for FS, PT, EC, and PD are provided in Table 1. Overall, there were no statistically significant correlations (all *p*s > 0.06) (Fig. 5). Results from Cronbach’s *α* were as follows: Fantasy Scale (7 items) *α* = 0.76; Perspective Taking *α* = 0.79; Personal Distress (7 item) *α* = 0.78; and Empathic Concern (7 item) *α* = 0.73.

**Table 1:** Mean and SD of correlations between IRI and DSFMs performance index

	Mean	SD	N
PT	71.99	12.12	101
FS	69.50	13.08	101
EC	77.85	10.98	101
PD	59.44	13.56	101

### Correlation between identity and expressions recognition performances with/without DSFMs

Pearson’s correlations between memory (test with and without DSFMs during learning) and emotion (five emotions with and without DSFMs) performance have been run. Results, with alpha corrected for multiple comparisons, showed no statistically significant correlation between emotion and identity performances (all *p*s > 0.008, corrected alpha = 0.002), thus suggesting that the two processes are unrelated and differentially affected by DSFMs.

## Discussion

The COVID-19 pandemic has drastically changed the way we relate to each other (Singh and Singh 2020) since isolation and DSFMs have significantly impacted human social interactions (Melendez et al. 2020). When people wear DSFMs, the bottom-half of their face (i.e., nose tip, mouth, overall face contour) is covered, and the available perceptual information is reduced. Faces are also the major communication channel for emotion expression (Armony and Vuilleumier 2013). As a matter of fact, emotion expression recruits face muscles in unique ways, and some areas of the face—mainly the eyes, nose, and mouth—convey different fundamental cues (Ekman 1993; Shiota et al. 2003). With safety devices covering about 60–70% of the face area that is relevant for emotional expression, emotions recognition gets harder (Grundmann et al. 2021; Marini et al. 2021). Given the massive use of DSFMs in daily life during the COVID-19 pandemic, understanding the mechanism of compromised identity recognition and emotional perception is of considerable importance (Grundmann et al. 2021).

As such, our study had two aims: firstly, to investigate to what extent DSFMs affect recognition, and whether face learning conditions (presence/absence of DSFMs) could affect recognition performance; secondly, we wanted to assess whether DSFMs interfere with facial emotion recognition, and if high empathy levels could facilitate emotion recognition in faces wearing DSFMs.

### Facial identity recognition and DSFMs

The upper half of the face, especially the eyes, allows people to properly recognize faces (Dal Martello and Maloney 2006; Fisher and Cox 1975). However, the lower part also plays an important role in this process, as pointed out from previous research showing that mouth covering (as in the case of DSFMs) leads to a reduced recognition accuracy compared to unobstructed faces, as a result of the interferences with holistic processes (Tanaka and Farah 1993; Tanaka and Sengco 1997). Indeed, the observer is no longer able to process key information about spatial relationships between facial features (Maurer et al. 2002). On this point, our study shows that in some cases DSFMs have a strong effect on performances, specifically when faces are shown and learned partially covered (learning condition with DSFMs). Indeed, in Block 1, in which the learning phase took place without DSFMs, a reduction in face discrimination performances occurred with DSFMs. By contrast, in Block 2, with the learning phase including only faces with DSFMs, participants were better at discriminating faces with than without DSFMs. One plausible explanation of face recognition worsening in Block 1 is that lower face coverage could have disrupted holistic face processing, thus making it difficult for the observer to extrapolate the configural information, and to elaborate a unified representation of the face when an obstacle was present (Carragher 2020).

In Block 2, participants were asked to memorize faces with DSFMs, therefore holistic processing might not take place. We can speculate that, in this case, feature-based processes were immediately engaged, and the observers’ focus moved to the individual characteristics of the observed face (Tanaka and Farah 1993); thus, DSFMs hindered face processing as a unique configuration. Despite face recognition relying on both featural and configural processing, their effects are sometimes dissociable (Cabeza and Kato 2000). Indeed, several studies show the importance of featural aspects. For instance, Kimchi and Amishav (2010) showed that when faces differ in one component only (e.g., eyes, nose, or mouth), correct discrimination between similar identities is determined by the discriminability of that component itself. Similarly, Cabeza and Kato (2000) showed that similar individual features between learned target faces and new different ones tend to impair identity recognition (i.e., defined as the “prototype effect”). This suggests that faces’ individual components, if available in memory, can guide face recognition. This is what probably happened in our study when faces were partially occluded by the DSFMs, with holistic processing overtaken by featural recognition. Moreover, previous studies in which participants were asked to memorize face parts (e.g., nose or eyes) showed that it is hard to ignore irrelevant information when the learned parts are embedded in a full face (i.e., holistic interference), while the performance is good when participants are asked to recognize single parts only (Leder and Carbon 2005). These findings are in line with our results, where better recognition for faces wearing DSFMs only emerged if faces were previously learnt with DSFMs. In everyday life, we might have all experienced this phenomenon during the COVID-19 pandemic, when, after meeting new people with the DSFMs, we were surprised to see “how their faces looked like” as soon as seen after the DSFMs were taken down for the first time.

The relevance of the learning modality in face memory emerged also in terms of response bias (i.e., participants’ willingness to respond that a target face in the testing phase did appear in the previous learning phase). Indeed, we found that participants’ responses were more conservative (i.e., higher tendency to reject the “target”) when DSFMs were absent and the study faces were learnt without DSFMs. By contrast, when faces were learnt with DSFMs, responses were more conservative in trials with DSFMs. This result highlights a stronger conservative response bias when the learning modality matched the test modality, with higher caution toward the risk for false alarms. The fact that performances appear to depend upon the learning stage could reflect separate processing tracks for faces learnt with and without DSFMs.

Overall, our results from Experiment 1 suggest that (i) DSFMs have an overall detrimental effect on memory performance but, critically, (ii) this effect is mediated by learning (with or without DSFMs). This also indicates that (iii) masked and unmasked face processing might rely on qualitatively different mechanisms; specifically, holistic processing represents the “default mode” of face processing, whereas under certain conditions (e.g., masked faces) face recognition could be achieved with featural processing.

However, even though our results are in line with Bruce and Young’s (1986) theory of facial processing, which indicates that any odd element in partially covered faces interferes with the proper face structural encoding process (which normally takes place with a totally uncovered face), and there is evidence of DSFMs disrupting holistic processing in some recent work through face inversion effect (Freud et al. 2020; Stajudhar et al. 2022), we did not directly test holistic processes, and thus, we cannot exclude that the observed effects might be due to a general (i.e., non-face sensitive) context effect.

### Facial expression recognition and DSFMs

The debate on the processes underlying emotional facial expressions recognition is still ongoing. Some theories stress the importance of holistic processes (Tanaka et al. 2012; Prazak and Burgund 2014; White 2000), while others emphasize specific facial features’ role (Calvo and Nummenmaa 2008; Ellison and Massaro 1997). Specifically, studies using different experimental manipulations supported the role of holistic and configural mechanisms in the recognition of facial expressions, as evidenced by the face inversion (Derntl et al. 2009a, b; Prkachin 2003) and composite (Calder and Janesen 2005) effects, two paradigms designed to specifically that impair holistic face processing.

On the contrary, other evidence suggests that emotion recognition is based on individual facial features (e.g., pulling the corners of the mouth or lowering the eyebrows) (Calvo and Nummenmaa 2008), as also emerged from eye-tracking studies (Bombari et al. 2013). Since the specific features of each emotion are heterogeneously distributed across the face (Eisenbarth and Alpers 2011), people tend to preferentially look at the features that are peculiar to each emotion (Calvo and Nummenmaa 2008). It has been shown that, under certain conditions, people rely more on feature-based mechanisms of emotion recognition rather than holistic ones, as in the case of prosopagnosia (Palermo et al. 2011). It is therefore possible that, given the different and complex characteristics of each emotion, recognition cannot be reduced to features processing or holistic processing alone for all emotions (Beaudry et al. 2013).

We hypothesized that the DSFMs would have determined an overall decrease in emotion recognition, in line with recent findings on face and emotion perception with DSFMs (Grundmann et al. 2021; Marini et al. 2021). However, DSFMs in our study had specific effects on different emotions. This result could stem from the characteristic traits of each emotion, as well as their related recognition processes (Wegrzyn et al. 2017). No differences emerged in our study for *fear* recognition with and without DSFMs, which is in line with previous studies showing it mainly relies on the upper area of the face (i.e., the eyes) (Beaudry et al. 2013), which typically appear open and tense, firm on a fixed point (Ekman and Friesen 2003). In line with our results, fear is conveyed by high-, but not low-, spatial frequencies, and should not be affected by nose and mouth covering (Smith and Schyns 2009). A similar result emerged for *anger* recognition, with no significant differences between the two conditions. A recent study (Grenville and Dwyer 2022), paradoxically, showed that anger recognition accuracy was higher with DSFMs compared to faces with no DSFMs. This result, which has been replicated in our data based on accuracy (see Supplementary material), might stem from biased participants’ responses; as highlighted by SDT (*d’*) analysis, anger discrimination was not actually better with DSFMs, but it was simply signaled more frequently as “target present,” thus increasing the possibility to collect a higher number of correct answers. The literature on anger and fear recognition reports a recognizable-top bias, which means that it relies heavily on information from the upper half of the face, most likely the eye region (Calder et al. 2000; Wegrzyn et al. 2015).

With respect to *happiness*, discrimination got worse with DSFMs. This emotion is mostly recognized through the mouth; however, since it is the only “pure” positive emotion and occurs more often than sadness, fear, or anger in human relationships (Tomkins 1962), it could be easier to recognize happy faces even if the lower part of the face is obscured. The eyes, therefore, seeme sufficient for happiness recognition in most of the cases, even if holistic processes were disrupted by DSFMs. As happened for happiness, discriminability for faces expressing *sadness* was significantly worse with DSFMs. Although sadness’ peculiar features are drawn-down lips corners and lowered and knitted eyebrows, this should not be a distinctive sadness feature, since it is shared with anger and fear. It is therefore possible that DSFMs led to worse recognition performances due to the disrupted holistic processes (i.e., the matching of eyes with mouths).

Lastly, *disgust* recognition was the most affected by DSFMs presence, switching from being the best discriminated emotion without the DSFMs to being the least discriminated with DSFMs. In this case, the idiosyncratic trait of reference is the mouth, which also conveys the intensity level of the emotional state experienced (Ekman and Friesen 2003). Given that accurate recognition requires focusing on the lower part of the face, participants’ disgust discrimination performances worsened when this was obscured by the DSFMs (Beaudry et al. 2013).

Confusion matrices (see Supplementary Figs. 1 and 2) could also help us to highlight potential systematic errors’ patterns between the different emotions. The emotion that has been much affected by DSFMs was disgust; it could be thus interesting to look more deeply at disgust misclassification pattern pointed out by the confusion matrices (Supplementary Figs. 1 and 2), which show that disgust was sometimes misperceived as anger compared to other emotions when faces were completely available (Supplementary Fig. 1), but it was mostly labeled as anger when faces were occluded by DSFMs (Supplementary Fig. 2); however, the contrary did not happen (i.e., anger was less misperceived as disgust). This qualitative misinterpretation between anger and disgust has already been documented in other studies (Wegrzyn et al. 2017; Pochedly et al. 2012), and interpreted on the basis of the “nose scrunch” feature shared by both these emotions (Pochedly et al. 2012), as well as the pulled down eyebrows (Dubey and Singh 2016). Thus, when the diagnostic feature of disgust (i.e., mouth) is hidden, people could have the tendency to base their judgment mainly on the eyes, which is similar to that of anger expressions, and erroneously recognize disgust as anger.

Further mechanisms might account for our results, such as the participants’ emotional state, and their psychological and social condition, that are capable of impacting face recognition abilities (Alharbi et al. 2019) (e.g., isolation during the pandemic). As such, the drastic and “negative” changes in daily life habits could have fostered people’s psychological distress, and even caused symptoms of post-traumatic stress disorder (PTSD) or mood alteration, even in the healthy population (Bai et al. 2004; Brooks et al. 2020). Social confinement, as in the time of the COVID-19 pandemic, might induce people to focus on stimuli having a negative component (e.g., sadness). This is explained by the “emotional congruence” phenomenon (Meléndez et al. 2020), which postulates that emotional states tend to ease the encoding of stimuli having the emotional valence the encoder is experiencing (Loeffler et al. 2013). Moreover, when individuals are exposed to stressful situations (that is the case of a global pandemic), they tend to develop anger as a reactive means of establishing safety (Smith et al. 2021). In addition, angry faces broadly represent an important cue of social threat, and several experiments showed that they are detected more accurately, and require shorter processing time and fewer attentional resources as compared to other emotions (Pinkham et al. 2010; Calvo et al. 2006). The potential adaptive value of anger recognition could further explain our findings about participants’ tendencies to misinterpret disgust as anger.

Our results from response bias show that participants’ biased tendencies varied based on the specific emotion and the DSFMs Vs. no DSFMs condition. Indeed, while no significant bias emerged for happiness and fear, participants showed conservative tendencies (i.e., a higher threshold for judging that a certain emotion was present) for stimuli without DSFMs expressing anger and sadness than with DSFMs. By contrast, when the target emotion was disgust, participants’ bias was more conservative with DSFMs than without. However, every erroneous response for each emotion in our task might have differentially impacted the others (e.g., a high rate of false alarms for the target anger impact hits, misses, and correct rejections in a different way for each other emotion), which in turn could affect response bias scores. When considering the responses for each alternative (in our case, each target emotion with and without DSFMs), we should take into account that responses are not “independent” from each other. Indeed, we could assume participants were more (or less) prone to the risk for erroneous responses with certain emotions than with others, but it would be hard to completely disentangle emotions’ “cross-sectional” effects. To facilitate results’ interpretation, it is possible to consider the confusion matrices (see Supplementary material) revealing the relationship between each target with and without DSFMs; for example, anger happens to be the higher chosen response with DSFMs compared to other emotions. The rates of confusion highlight the non-independence of our target’s levels and imply caution when interpreting SDT response bias.

Overall, our results suggest that DSFMs affect recognition processes of some specific emotions, and this might point out a possible complementary, flexible, and interactive role of holistic and feature-based processes in emotion recognition, especially in atypical situations, as when diagnostic information is limited to a specific face region and cannot be based on the whole face. We can speculate that the DSFMs operate as a “misaligning condition” (i.e., top and bottom halves of the face are spatially misaligned) as seen in the “Composite-face task” (Calder et al. 2000). As such, it interferes with the holistic processes commonly used to recognize emotions when the entire face is available (Tanaka et al. 2012). When the DSFM is on, local processes need to be engaged, leading to an alteration (potentially) of facial expression processing. Since emotions have their peculiar characteristics, but at the same time they share features (e.g., nose “scrunch” in anger and disgust), we might argue that holistic but not feature-based processing is advantageous for the recognition of some emotions, while feature-based but not holistic processing is a preferential strategy for other expressions.

### Emotion recognition and empathy

The present study also aimed to investigate a potential correlation between empathy levels and emotion recognition with DSFMs. Since previous studies demonstrated positive correlations between empathy scores and emotion recognition (Gery et al. 2009; Besel and Yuille 2010), we hypothesized that higher empathy levels could facilitate emotion recognition even when DSFMs cover a large part of the face. However, contrary to our expectations, no significant results emerged from the correlations between DSFMs Performance Indexes and the IRI’s different subscales. A study from Ramachandra and Longacre (2022) showed that people who are more empathetic exhibit better recognition performances for “eyes-only” emotions (i.e., similar to masked facial expressions) than others. Our results’ divergence from this recent evidence could be discussed in light of some methodological differences; for instance, authors adopted pictures of the “upper part” of a face (i.e., not faces wearing DSFM), with double choice trials of two “half-faces” expressing different emotions from the same identity throughout the entire experiment. We believe our study included stimuli with higher ecological validity (i.e., multiple identities which were not just cut but “digitally” masked). Also, the different questionnaires adopted to measure participants’ empathy could account for results’ divergence between the two studies. Moreover, we point out that the measurement of empathy through self-report questionnaires can unveil limited facets of individuals’ empathy (Losoya and Eisenberg 2001). Given the lack of research on how empathy mediates emotion recognition with and without masks, future studies should further investigate this relevant social implication of DSFMs, potentially via multimodal approaches to obtain greater comprehensive assessments of individuals’ empathy (Zhou et al. 2003). In conclusion, we highlight that emotion perception is a complex process with multiple aspects (other than empathy) potentially impacting on participants’ recognition rates, and mediating its effects on people’s performances (e.g., gender, age, typical/pathological individual differences) (Pazhoohi et al. 2021; Palmisano et al. 2021; Sedda et al. 2013).

### Relationship between identity and emotions with and without DSFMs

Our results from correlations between the old-new face memory task and facial affect task showed non-significant relationships between performances at the two tasks with and without DSFMs. Although previous literature suggests that the processing of identity and expression diverges at an early stage of face perception, which would imply a weak or no correlation between the two (Haxby et al. 2000; Palermo et al. 2013), some authors refer to emotion recognition and face identity recognition as related independent constructs (Connolly et al. 2020) sharing a partially common ability, independent from intelligence and short-term memory (Connolly et al. 2019). Our results provide evidence for a dissociable effect of DSFMs on memory and emotion recognition, in line with a recent study showing  no correlation between familiar face matching and emotion categorization (Noyes et al. 2021). Thus, we can speculate that face recognition and emotion perception are unrelated and differentially affected by DSFMs. The inconsistency between our results and some previous studies might be based on methodological differences (e.g., tasks, experimental paradigms).

### Conclusion, limitations, and future directions

Overall, our data indicate that DSFMs negatively affect both human memory for unfamiliar faces and facial expression recognition. This effect, however, is not linear since it interacts with other factors. Specifically, identity recognition is not affected by DSFMs *only* when faces are also learned with DSFMs, highlighting that the recognition process might depend on the learning context. This also emphasizes how our brain is able to adapt to context and situations; therefore, we can only make speculation about the behavioral and neuroplastic effects of long-term use of DSFMs (Costantino et al. 2017). In addition, a general worsening of emotion recognition with DSFMs was found for all emotions except anger. Concerning empathy, our study found no significant correlation between empathy and emotion recognition. Considering that identity and emotion recognition processes play a fundamental role in social interactions, future research should focus on how humans will adapt to the challenging environmental conditions of our times while safeguarding their primary need for affective communication.

This study has some potential limitations. Firstly, the online administration of the tasks implies reduced control over the experimental conditions. Secondly, our study was conducted on a heterogeneous but not fully representative sample concerning age and sex in the general population. Moreover, as the sample was composed of Italian people only, various cultural influences cannot be excluded. Keeping external face features (such as hair) in the task stimuli could be another potential limit, by facilitating face recognition. In addition, we did not measure participants’ satisficing or inattention when completing the self-report questionnaire. Finally, the adoption of a multiple-choice task in Experiment 2 implies that results’ intelligibility in terms of response bias is limited, although considering confusion matrices (Ingleby 1973). Future studies would expand our analyses and results to clarify how the massive use of DSFMs affects social interactions and unravel its social and psychological effects.

## Supplementary Information

Below is the link to the electronic supplementary material.

Supplementary file1 (DOCX 110 kb)

## Footnotes

1 The calculation was performed with the following criteria: effect size f = 0.253 (which was derived from a medium partial eta squared of η_p_^2^ = 0.06); α = .05; power = .95; number of groups = 1; correlation among repeated measures = 0.5 (default); non-sphericity correction = 1 (default). The number of measurements changed between Experiment 1 (= 4) and Experiment 2 (= 10) (Faul et al. 2007).

## References

[CR1] Albiero P, Ingoglian S, Lo Coco A (2006). Contributo all’adattamento italiano dell’Interpersonal Reactivity Index. Test Psicometria Metodol.

[CR2] Alharbi J, Jackson D, Usher K (2019). Compassion fatigue in critical care nurses. Saudi Med J.

[CR3] Armony J, Vuilleumier P (eds) (2013) *The Cambridge handbook of human affective neuroscience.* pp xii, 667. Cambridge University Press. 10.1017/CBO9780511843716

[CR4] Bai Y, Lin C-C, Lin C-Y, Chen J-Y, Chue C-M, Chou P (2004). Survey of stress reactions among health care workers involved with the SARS outbreak. Psychiatr Serv.

[CR5] Beaudry O, Roy-Charland A, Perron M, Cormier I, Tapp R (2013). Featural processing in recognition of emotional facial expressions. Cogn Emot.

[CR6] Besel LDS, Yuille JC (2010). Individual differences in empathy: the role of facial expression recognition. Personal Individ Differ.

[CR7] Bobak AK, Dowsett AJ, Bate S (2016). Solving the border control problem: evidence of enhanced face matching in individuals with extraordinary face recognition skills. PLoS ONE.

[CR8] Bombari D, Schmid PC, Mast MS, Birri S, Mast FW, Lobmaier JS (2013). Emotion recognition: the role of featural and configural face information. Quart J Exp Psychol.

[CR9] Bonemei R, Costantino AI, Battistel I, Rivolta D (2018). The perception of (naked only) bodies and faceless heads relies on holistic processing: Evidence from the inversion effect. Br J Psychol.

[CR120] Bossi F, Premoli I, Pizzamiglio S, Balaban S, Ricciardelli P, Rivolta D (2020). Theta-and gamma-band activity discriminates face, body and object perception. Front Hum Neurosci.

[CR10] Brooks SK, Webster RK, Smith LE, Woodland L, Wessely S, Greenberg N, Rubin GJ (2020). The psychological impact of quarantine and how to reduce it: Rapid review of the evidence. Lancet.

[CR11] Bruce V, Young A (1986). Understanding face recognition. Br J Psychol.

[CR12] Bull FC, Al-Ansari SS, Biddle S, Borodulin K, Buman MP, Cardon G, Carty C, Chaput J-P, Chastin S, Chou R, Dempsey PC, DiPietro L, Ekelund U, Firth J, Friedenreich CM, Garcia L, Gichu M, Jago R, Katzmarzyk PT, Willumsen JF (2020). World Health Organization 2020 guidelines on physical activity and sedentary behaviour. Br J Sports Med.

[CR13] Burton AM, White D, McNeill A (2010). The glasgow face matching test. Behav Res Methods.

[CR400] Cabeza R, Kato T (2000). Features are also important: contributions of featural and configural processing to face recognition. Psychol Sci.

[CR14] Cacioppo JT, Ernst JM, Burleson MH, McClintock MK, Malarkey WB, Hawkley LC, Kowalewski RB, Paulsen A, Hobson JA, Hugdahl K, Spiegel D, Berntson GG (2000). Lonely traits and concomitant physiological processes: the MacArthur social neuroscience studies. Int J Psychophysiol.

[CR15] Calbi M, Langiulli N, Ferroni F (2021). The consequences of COVID-19 on social interactions: an online study on face covering. Sci Rep.

[CR16] Calder AJ, Young AW, Keane J, Dean M (2000). Configural information in facial expression perception. J Exp Psychol Hum Percept Perform.

[CR17] Calder AJ, Jansen J (2005). Configural coding of facial expressions: the impact of inversion and photographic negative. Vis Cogn.

[CR18] Calvo MG, Avero P, Lundqvist D (2006). Facilitated detection of angry faces: initial orienting and processing efficiency. Cogn Emot.

[CR19] Calvo MG, Nummenmaa L (2008). Detection of emotional faces: salient physical features guide effective visual search. J Exp Psychol Gen.

[CR20] Carragher DJ, Hancock PJB (2020). Surgical face masks impair human face matching performance for familiar and unfamiliar faces. Cognit Res Princ Implic.

[CR21] Connolly HL, Young AW, Lewis GJ (2019). Recognition of facial expression and identity in part reflects a common ability, independent of general intelligence and visual short-term memory. Cogn Emot.

[CR22] Connolly HL, Lefevre CE, Young AW, Lewis GJ (2020). Emotion recognition ability: evidence for a supramodal factor and its links to social cognition. Cognition.

[CR890] Costantino AI, Titoni M, Bossi F, Premoli I, Nitsche MA, Rivolta D (2017). Preliminary evidence of “Other-Race Effect”-like behavior induced by Cathodal-tDCS over the right occipital cortex, in the absence of overall effects on Face/Object processing. Front Neurosci.

[CR23] Cowan DG, Vanman EJ, Nielsen M (2014). Motivated empathy: The mechanics of the empathic gaze. Cogn Emot.

[CR24] Dal Martello MF, Maloney LT (2006). Where are kin recognition signals in the human face?. J vis.

[CR25] Davies G, Flin R (1984). The man behind the mask—Disguise and face recognition. Hum Learn J Pract Res Appl.

[CR26] Davis MH (1980). A multidimensional approach to individual differences in empathy. JSAS Catalog Select Doc Psychol.

[CR27] Davis MH (1983). The effects of dispositional empathy on emotional reactions and helping: a multidimensional approach. J Pers.

[CR28] Davis MH, Luce C, Kraus SJ (1994). The heritability of characteristics associated with dispositional empathy. J Pers.

[CR29] Decety J, Jackson PL (2006). A social-neuroscience perspective on empathy. Curr Dir Psychol Sci.

[CR30] Derntl B, Finkelmeyer A, Toygar TK, Hülsmann A, Schneider F, Falkenberg DI, Habel U (2009). Generalized deficit in all core components of empathy in schizophrenia. Schizophr Res.

[CR31] Derntl B, Seidel E-M, Kainz E, Carbon C-C (2009). Recognition of emotional expressions is affected by inversion and presentation time. Perception.

[CR32] Dubey M, Singh PL (2016). Automatic emotion recognition using facial expression: a review. Int Res J Eng Technol (IRJET).

[CR33] Duchaine B, Nakayama K (2006). The Cambridge face memory test: results for neurologically intact individuals and an investigation of its validity using inverted face stimuli and prosopagnosic participants. Neuropsychologia.

[CR34] Duchaine BC, Parker H, Nakayama K (2003). Normal recognition of emotion in a prosopagnosic. Perception.

[CR35] Durand K, Gallay M, Seigneuric A, Robichon F, Baudouin J-Y (2007). The development of facial emotion recognition: the role of configural information. J Exp Child Psychol.

[CR36] Eisenbarth H, Alpers GW (2011). Happy mouth and sad eyes: scanning emotional facial expressions. Emotion.

[CR37] Ekman P (1992). Are there basic emotions?. Psychol Rev.

[CR38] Ekman P (1993). Facial expression and emotion. Am Psychol.

[CR450] Ekman P, Friesen WV (2003) Unmasking the Face. A Guide to Recognizing Emotions from Facial Clues. Malor Books, Los Altos, CA

[CR39] Ellison J, Massaro D (1997). Featural evaluation, integration, and judgment of facial affect. J Exp Psychol Hum Percept Perform.

[CR40] Farah MJ, Tanaka JW, Drain HM (1995). What causes the face inversion effect?. J Exp Psychol Hum Percept Perform.

[CR41] Faul F, Erdfelder E, Lang A-G, Buchner A (2007). G*Power 3: a flexible statistical power analysis program for the social, behavioral, and biomedical sciences. Behav Res Methods.

[CR42] Ferrari C, Vecchi T, Sciamanna G, Blandini F, Pisani A, Natoli S (2021). Facemasks and face recognition: potential impact on synaptic plasticity. Neurobiol Dis.

[CR43] Fisher GH, Cox RL (1975). Recognizing human faces. Appl Ergon.

[CR44] Freud E, Stajduhar A, Rosenbaum RS, Avidan G, Ganel T (2020). The COVID-19 pandemic masks the way people perceive faces. Sci Rep.

[CR45] Freud E, Stajduhar A, Rosenbaum R, Avidan G, Ganel T (2021). Recognition of masked faces in the era of the pandemic: No improvement, despite extensive, natural exposure. PsyArXiv (preprint).

[CR46] Gardner RM, Dalsing S, Reyes B (1984). Table of criterion values (*β*) used in signal detection theory. Behav Res Methods Instrum Comput.

[CR47] Garrido L, Duchaine B (2007). Do facial identity and facial expression processing dissociate in prosopagnosia? [Abstract]. J vis.

[CR48] Gery I, Miljkovitch R, Berthoz S, Soussignan R (2009). Empathy and recognition of facial expressions of emotion in sex offenders, non-sex offenders and normal controls. Psychiatry Res.

[CR49] Gori M, Schiatti L, Amadeo MB (2021). Masking emotions: face masks impair how we read emotions. Front Psychol.

[CR50] Graham DL, Ritchie KL (2019). Making a spectacle of yourself: the effect of glasses and sunglasses on face perception. Perception.

[CR51] Grenville E, Dwyer DM (2022). Face masks have emotion-dependent dissociable effects on accuracy and confidence in identifying facial expressions of emotion. Cognit Res Princ Implic.

[CR700] Green DM, Swets JA (1966) Signal Detection Theory and Psychophysics. Wiley, New York

[CR52] Grundmann F, Epstude K, Scheibe S (2021). Face masks reduce emotion-recognition accuracy and perceived closeness. PLoS ONE.

[CR53] Hall JA, Matsumoto D (2004). Gender differences in judgments of multiple emotions from facial expressions. Emotion.

[CR54] Haxby JV, Hoffman EA, Gobbini MI (2000). The distributed human neural system for face perception. Trends Cogn Sci.

[CR9000] Ingleby JD (1973). The separation of bias and sensitivity in multiple-choice tasks. Perception.

[CR345] Kapwing (2021). https://www.kapwing.com/explore/maskon-yourprofile-picture-for-covid19-safety

[CR55] Keltner D, Shiota M (2003). New displays and new emotions: a commentary on Rozin and Cohen (2003). Emotion.

[CR56] Kimchi R, Amishav R (2010). Faces as perceptual wholes: the interplay between component and configural properties in face processing. Vis Cogn.

[CR57] Kotsia I, Buciu I, Pitas I (2008). An analysis of facial expression recognition under partial facial image occlusion. Image vis Comput.

[CR58] Kramer RSS, Ritchie KL (2016). Disguising superman: how glasses affect unfamiliar face matching. Appl Cogn Psychol.

[CR600] Kret ME, de Gelder B (2012). Islamic headdress influences how emotion is recognized from the eyes. FrontPsychol.

[CR59] Leder H, Carbon C-C (2005). When context hinders! Learn-test compatibility in face recognition. Quart J Exp Psychol Hum Exp Psychol.

[CR60] Loeffler SN, Myrtek M, Peper M (2013). Mood-congruent memory in daily life: evidence from interactive ambulatory monitoring. Biol Psychol.

[CR61] Losoya SH, Eisenberg N, Hall JA, Bernieri FJ (2001). Affective empathy. Interpersonal sensitivity: theory and measurement.

[CR62] Lundqvist D, Flykt A, Öhman A (1998) The karolinska directed emotional faces - KDEF, CD ROM from Department of Clinical Neuroscience, Psychology section, Karolinska Institutet, ISBN 91-630-7164-9

[CR63] Ma DS, Correll J, Wittenbrink B (2015). The Chicago face database: a free stimulus set of faces and norming data. Behav Res Methods.

[CR64] Makowski D (2018). The psycho package: an efficient and publishing-oriented workflow for psychological science. J Open Source Softw.

[CR65] Manley KD, Chan JCK, Wells GL (2019). Do masked-face lineups facilitate eyewitness identification of a masked individual?. J Exp Psychol Appl.

[CR66] Mansour JK, Beaudry JL, Bertrand MI, Kalmet N, Melsom EI, Lindsay RCL (2020). Impact of disguise on identification decisions and confidence with simultaneous and sequential lineups. Law Hum Behav.

[CR67] Marini M, Ansani A, Paglieri F, Caruana F, Viola M (2021). The impact of facemasks on emotion recognition, trust attribution and re-identification. Sci Rep.

[CR800] Maurer D, Grand RL, Mondloch CJ (2002). The many faces of configural processing. Trends Cogn Sci.

[CR68] Mckone E, Crookes K, Kanwisher N (2009) The cognitive and neural development of face recognition in humans. In: *The cognitive neurosciences*, 4th ed. pp 467–482. Massachusetts Institute of Technology

[CR69] Meléndez JC, Satorres E, Reyes-Olmedo M, Delhom I, Real E, Lora Y (2020). Emotion recognition changes in a confinement situation due to COVID-19. J Environ Psychol.

[CR70] Monti C, Sozzi M, Bossi F, Corbo M, Rivolta D (2019). Atypical holistic processing of facial identity and expression in a case of acquired prosopagnosia. Cogn Neuropsychol.

[CR220] Negrini M, Brkić D, Pizzamiglio S, Premoli I, Rivolta D (2017). Neurophysiological correlates of featural and spacing processing for face and non-face stimuli. Front Psychol.

[CR71] Noyes E, Davis JP, Petrov N, Gray KL, Ritchie KL (2021). The effect of face masks and sunglasses on identity and expression recognition with super-recognizers and typical observers. R Soc Open Sci.

[CR72] Palermo R, Willis ML, Rivolta D, McKone E, Wilson CE, Calder AJ (2011). Impaired holistic coding of facial expression and facial identity in congenital prosopagnosia. Neuropsychologia.

[CR73] Palermo R, O’Connor KB, Davis JM, Irons J, McKone E (2013). New tests to measure individual differences in matching and labelling facial expressions of emotion, and their association with ability to recognise vocal emotions and facial identity. PLoS ONE.

[CR670] Palmisano A, Bossi F, Barlabà C, Febbraio F, Loconte R, Lupo A, Nitsche MA, Rivolta D (2021). Anodal tDCS effects over the left dorsolateral prefrontal cortex (L-DLPFC) on the rating of facial expression: evidence for a gender-specific effect. Heliyon.

[CR74] Patterson KE, Baddeley A (1977). When face recognition fails. J Exp Psychol Hum Learn Mem.

[CR75] Pazhoohi F, Forby L, Kingstone A (2021). Facial masks affect emotion recognition in the general population and individuals with autistic traits. PLoS ONE.

[CR76] Pinkham AE, Griffin M, Baron R, Sasson NJ, Gur RC (2010). The face in the crowd effect: anger superiority when using real faces and multiple identities. Emotion.

[CR77] Pochedly JT, Widen SC, Russell JA (2012). What emotion does the “facial expression of disgust” express?. Emotion.

[CR78] Poerio GL, Totterdell P, Emerson L, Miles E (2015). Love is the triumph of the imagination: daydreams about significant others are associated with increased happiness, love and connection. Conscious Cogn.

[CR79] Poerio GL, Totterdell P, Emerson L, Miles E (2016). Helping the heart grow fonder during absence: daydreaming about significant others replenishes connectedness after induced loneliness. Cogn Emot.

[CR80] Prazak ER, Burgund ED (2014). Keeping it real: Recognizing expressions in real compared to schematic faces. Vis Cogn.

[CR81] Prkachin GC (2003). The effects of orientation on detection and identification of facial expressions of emotion. Br J Psychol.

[CR82] Ramachandra V, Longacre H (2022). Unmasking the psychology of recognizing emotions of people wearing masks: the role of empathizing, systemizing, and autistic traits. Personal Individ Differ.

[CR83] Reisenzein R, Horstmann G, Schützwohl A (2019). The cognitive-evolutionary model of surprise: a review of the evidence. Top Cogn Sci.

[CR84] Rivolta D, Woolgar A, Palermo R, Butko M, Schmalzl L, Williams MA (2014). Multi-voxel pattern analysis (MVPA) reveals abnormal fMRI activity in both the “core” and “extended” face network in congenital prosopagnosia. Front Hum Neurosci.

[CR85] Rizzolatti G, Craighero L, Changeux J-P, Damasio AR, Singer W, Christen Y (2005). Mirror neuron: a neurological approach to empathy. Neurobiology of human values.

[CR86] Savaskan E, Summermatter D, Schroeder C, Schächinger H (2018). Memory deficits for facial identity in patients with amnestic mild cognitive impairment (MCI). PLoS ONE.

[CR780] Sedda A, Rivolta D, Scarpa P, Burt M, Frigerio E, Zanardi G, Piazzini A, Turner K, Francione S, Lo Russo G, Bottini G (2013). Ambiguous emotion recognition in temporal lobe epilepsy: the role of expression intensity. Cogn Affect Behav
Neurosci.

[CR87] Singh J, Singh J (2020) COVID-19 and Its Impact on Society. Electr Res J Soc Sci Hum, **2**(I), Available at SSRN: https://ssrn.com/abstract=3567837

[CR88] Shapiro PN, Penrod S (1986). Meta-analysis of facial identification studies. Psychol Bull.

[CR89] Smith FW, Schyns PG (2009). Smile through your fear and sadness: transmitting and identifying facial expression signals over a range of viewing distances. Psychol Sci.

[CR90] Smith LE, Duffy B, Moxham-Hall V, Strang L, Wessely S, Rubin GJ (2021). Anger and confrontation during the COVID-19 pandemic: a national cross-sectional survey in the UK. J R Soc Med.

[CR91] Song J, Wang L, Wang W, Huang D-S, Jiang C, Bevilacqua V, Figueroa JC (2012). Eyebrow segmentation based on binary edge image. Intelligent computing technology.

[CR92] Stajduhar A, Ganel T, Avidan G (2022). Face masks disrupt holistic processing and face perception in school-age children. Cogn Res.

[CR93] Stanislaw H, Todorov N (1999). Calculation of signal detection theory measures. Behav Res Methods Instrum Comput.

[CR94] Steptoe A, Shankar A, Demakakos P, Wardle J (2013). Social isolation, loneliness, and all-cause mortality in older men and women. Proc Natl Acad Sci.

[CR95] Stoet G (2010). PsyToolkit: a software package for programming psychological experiments using Linux. Behav Res Methods.

[CR96] Tanaka JW, Farah MJ (1993). Parts and wholes in face recognition. Quart J Exp Psychol Sect A.

[CR97] Tanaka JW, Kaiser MD, Butler S, Grand RL (2012). Mixed emotions: Holistic and analytic perception of facial expressions. Cogn Emot.

[CR98] Tanaka JW, Sengco JA (1997). Features and their configuration in face recognition. Mem Cognit.

[CR99] Taylor MR, Agho KE, Stevens GJ, Raphael B (2008). Factors influencing psychological distress during a disease epidemic: Data from Australia’s first outbreak of equine influenza. BMC Public Health.

[CR100] Tomkins SS (1962) *Affect, imagery, consciousness, vol. 1: The positive affects*. Springer, New York

[CR101] Tottenham N, Hare TA, Casey BJ (2011). Behavioral assessment of emotion discrimination, emotion regulation, and cognitive control in childhood, adolescence, and adulthood. Front Psychol.

[CR102] Tracy JL, Matsumoto D (2008). The spontaneous expression of pride and shame: evidence for biologically innate nonverbal displays. Proc Natl Acad Sci.

[CR103] Wang B (2013). Gender difference in recognition memory for neutral and emotional faces. Memory.

[CR104] Wang Q, Chen G, Wang Z, Hu CS, Hu X, Fu G (2014). Implicit racial attitudes influence perceived emotional intensity on other-race faces. PLoS ONE.

[CR105] Webb SJ, Neuhaus E, Faja S (2017). Face perception and learning in autism spectrum disorders. Quart J Exp Psychol.

[CR106] Wegrzyn M, Vogt M, Kireclioglu B, Schneider J, Kissler J (2017). Mapping the emotional face. How individual face parts contribute to successful emotion recognition. PLoS ONE.

[CR107] Wegrzyn M, Bruckhaus I, Kissler J (2015). Categorical perception of fear and anger expressions in whole, masked and composite faces. PLoS ONE.

[CR108] White M (2000). Parts and Wholes in Expression Recognition. Cogn Emot.

[CR109] Widen SC, Russell JA (2003). A closer look at preschoolers’ freely produced labels for facial expressions. Dev Psychol.

[CR110] Zabelina DL, Zaonegina E, Revelle W, Condon DM (2021). Creative achievement and individual differences: Associations across and within the domains of creativity. Psychol Aesthet Creat Arts.

[CR111] Zabelina D, Clay J, Upshaw J (2021b) *in press*. Imagination, anxiety, and loneliness during the COVID-19 pandemic. 10.31234/osf.io/9aqbj

[CR112] Zhou Q, Valiente C, Eisenberg N (2003) Empathy and its measurement. In: Lopez SJ, Snyder CR (eds) Positive psychological assessment: A handbook of models and measures. pp 269–284. American Psychological Association. 10.1037/10612-017

