# Supplementary material for: Face memory and facial expression recognition are both affected by wearing disposable surgical face masks
Source: Cogn Process. 2022 Oct 15;24(1):43–57. doi: 10.1007/s10339-022-01112-2 (PMC9568966; doi:10.1007/s10339-022-01112-2)
Supplement: Supplementary file 1 — Supplementary file1 (DOCX 110 kb) [file 10339_2022_1112_MOESM1_ESM.docx]

1. **Supplementary methods**

*1.1 Experiment 1 – Old-new face memory task*

The data from the *Old-new face memory task* were analyzed using a repeated measures ANOVA with two factors: ‘learning’ (without DSFMs vs with DSFMs) and ‘test’ (face recognition without DSFMs vs with DSFMs).  Post-hoc analyses were Bonferroni corrected. All analyses were carried out using SPSS (Version 26.0).

- 1. *Experiment 2 – Facial affect task*

The data from the *Facial affect task* were analyzed using a repeated measures ANOVA including two factors: ‘emotion’ (disgust, fear, happiness, sadness, and anger) and ‘condition’ (without DSFM vs with DSFM). Post-hoc analyses were Bonferroni corrected. We created a Confusion Matrix to assess recognition accuracy and misclassification for each emotion specifically, both with and without DSFMs; the matrices were obtained by counting the correct classifications and the misclassifications during the recognition of emotions for all subjects. The values obtained express the number of identification and misinterpretation of a specific target, thus allowing us to perform a more insightful exploration of which emotion is more likely to be misclassified, and to specifically detect with which emotion it is mistaken.

1. **Supplementary results**

*2.1 Experiment 1 – Old-new face memory task*

The repeated measures ANOVA showed a main effect of “learning”, where learning without DSFMs (M = 81.6%, SEM = 1.2) led to a better performance than learning with DSFMs (M = 78.4%, SEM = 1.1) [F(1,100) = 6.08, *p* = .015]. There was no main effect of “test” [F(1,100) = 0.66, *p* = .42]. There was a statistically significant interaction between learning and test [F(1,100) = 172.23, *p* < .001]. Post-hoc comparisons indicate that recognition without DSFMs (M = 87.9%, SEM = 1.22) was higher than recognition with DSFMs (M = 75.3%, SEM = 1.56) when the study faces were presented *without* DSFMs, SEM = 1.43, *p* <.001 (Block 1). However, recognition without DSFMs (M = 71.3%, SEM = 1.36) was lower than recognition with DSFMs (M = 85.48%, SEM = 1.21) when the study faces were presented *with* DSFMs, SEM = 1.37, *p* <.001 (Block 2). These results, thus, demonstrate that it is easier for participants to recognize faces without DSFMs only if the same faces are learnt without DSFMs; contrary, they find it easier to recognize faces with DSFMs if they were exposed to them with DSFMs.

- 1. *Experiment 2 – Facial affect task*

The one-way repeated measures ANOVA showed a main effect of “emotion” [F(4,400) = 146.9, *p* <.001], where disgust recognition (M = 61.45%, SEM = 1.1) is lower than fear (M = 77.65% SEM = 1.3), happiness (M = 97%, SEM = 0.6), sadness (M = 69.66%, SEM = 1.5) and anger (M= 80.5%, SEM = 1.26); fear is worse recognized than happiness and anger, but it is better recognized than sadness; happiness recognition is higher than sadness and anger; finally, sadness recognition is lower than anger. There was a main effect of DSFMs [F(1,100) = 123.62, *p* <.001], showing that face emotions without DSFMs (M = 82.64%, SEM = 0.82) were better recognized than faces with DSFMs (M = 71.9%, SEM = 0.84).

There was also a statistically significant interaction between emotion and DSFMs [F(4,400) = 222, *p* <.001]. Post-hoc comparisons indicate that disgust recognition was higher without DSFMs (M= 91.8%, SEM = 1.1) than with DSFMs (M = 31% SEM = 2), SEM = 2.37, *p* <.001; similarly, happiness recognition was higher without DSFMs (M = 99%; SEM = 0.5) than with DSFMs (M = 95.1%; SEM =1.1), SEM = 1.15, *p* = .001, and sadness recognition was higher without DSFMs (M = 73.4%; SEM = 2) than with DSFMs (M = 65.9%, SEM = 1.8), SEM = 2.35, *p* = .002. Surprisingly, anger recognition was higher with DSFMs (M = 88.7%, SEM = 1.3) than without (M = 72.3%, SEM = 1.9), SEM = 2.02, *p <.*001. No statistically significant differences were found in fear recognition with (M = 78.7%, SEM = 1.8) and without (M = 76.6% SEM = 1.5) DSFMs, SEM = 1.97, *p* =.296.

Emotion identification errors analyzed by the confusion matrix for no DSFM condition (Figure 5) showed that disgust (91.7%) was more often mistaken for anger (5.9%) compared to the other emotion; fear (76.5%) was mixed up with disgust (13.7%); sadness (72.9%) was mostly mistaken for both disgust (8.7%) and fear (12.7%); anger (72.1%) for both disgust (10.9%) and fear (11%). Happiness was correctly labeled in almost all cases (99%). Regarding the DSFM condition (Figure 6), disgust (31.2%) was mistaken with anger over half of the time (43.2%); fear (78.9%) was confused with disgust (8%); sadness (65%) with fear (17%) and disgust (12%) and anger (88.6%) was sometimes labeled as disgust (6%); in this latter case however, anger’s identification rate was higher than no DSFM condition (88.6% vs 72.1%). Finally, happiness kept almost the same recognition rate of no DSFM condition (95%).

1. **Supplementary figures**

**
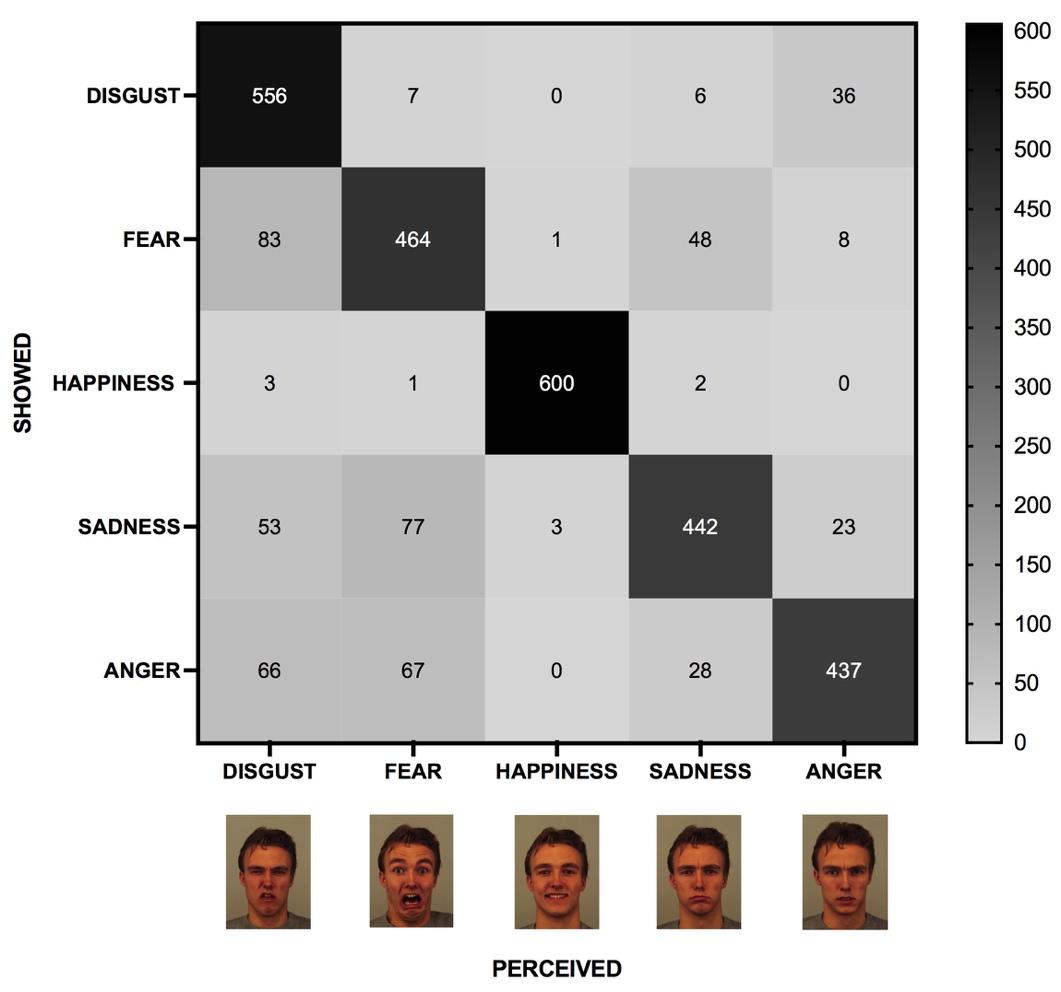
**

**Figure 1.** Heat map of the confusion matrix for the “without DSFMs” condition.

**
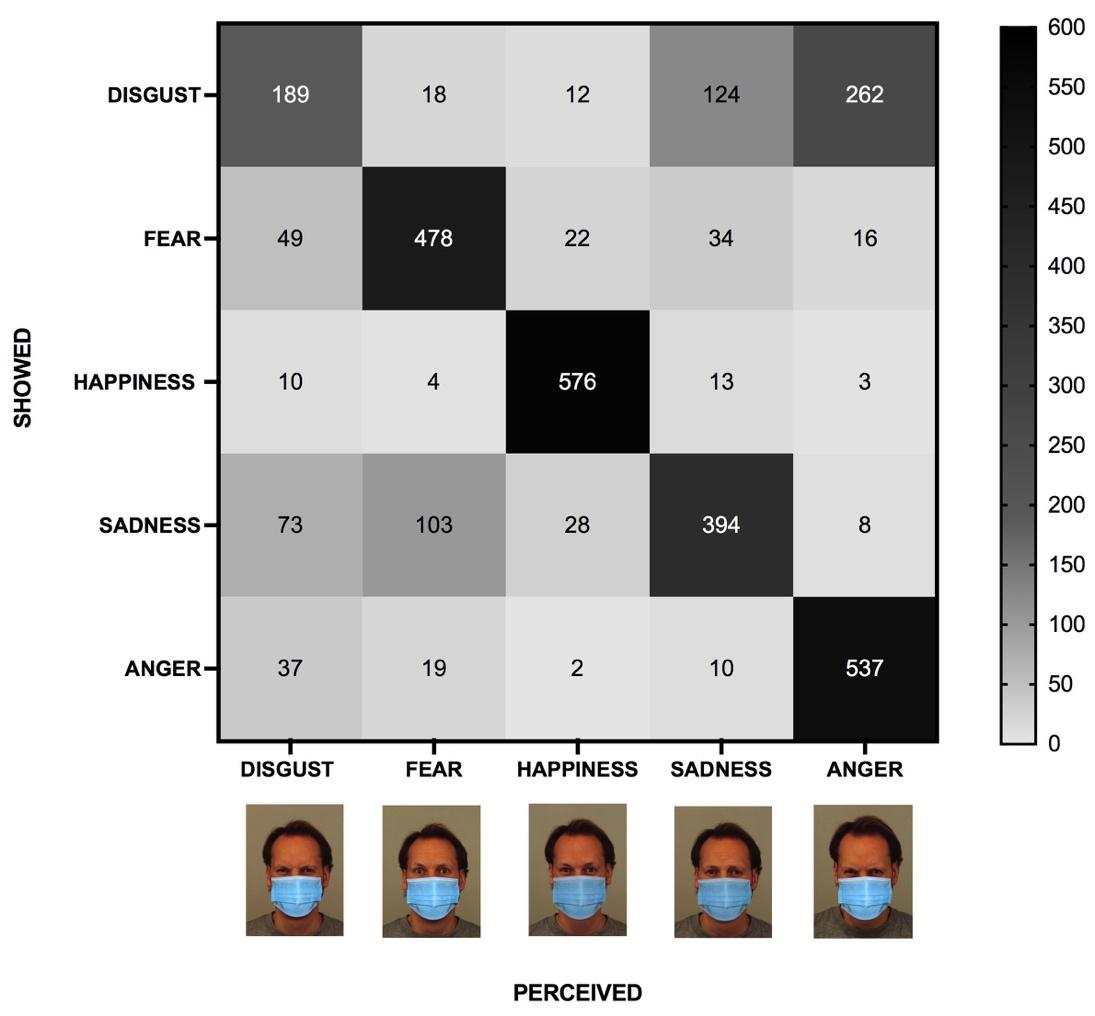
**

**Figure 2.** Heat map of the confusion matrix for the “with DSFMs” condition.
